# Untargeted Metabolite Profiling Reveals Acute Toxicity of Pentosidine on Adipose Tissue of Rats

**DOI:** 10.3390/metabo14100539

**Published:** 2024-10-09

**Authors:** Chuanqin Hu, Zhenzhen Shao, Wei Wu, Jing Wang

**Affiliations:** 1 School of Light Industry Science and Engineering, Beijing Technology and Business University (BTBU), 11 Fucheng Road, Beijing 100048, China; huchuanqin@btbu.edu.cn (C.H.);; 2 Key Laboratory of Geriatric Nutrition and Health, Beijing Technology and Business University, Ministry of Education, Beijing 100048, China

**Keywords:** food safety, mass spectrometry, metabolomics, pentosidine, untargeted metabolite profiling

## Abstract

**Background**: Pentosidine is an advanced glycation end product that is commonly found in heat-processed foods. Pentosidine has been involved in the occurrence and development of some chronic diseases. It was reported that pentosidine exposure can impair the function of the liver and kidneys. Adipose tissue, as an active endocrine organ, plays an important role in maintaining the normal physiological function of cells. However, the metabolic mechanism that causes pentosidine to induce toxicity in adipose tissue remains unclear. **Methods**: In the study, thirty male Sprague-Dawley rats were divided into a normal diet group, low dose group, and high dose group. A non-targeted metabolomics approach was used to compare the metabolic profiles of adipose tissue between the pentosidine and normal diet groups. Furthermore, histopathological observation and body weight change analysis were performed to test the results of the metabolomics analysis. **Results**: A total of forty-two differential metabolites were identified. Pentosidine mainly disturbed twelve metabolic pathways, such as ascorbate and aldarate metabolism, glycine, serine, and threonine metabolism, sulfur metabolism, pyruvate metabolism, etc. Additionally, pyruvic acid was identified as a possible key upregulated metabolite involved in thirty-four metabolic pathways. α-Ketoglutaric acid was named as a probable key downregulated metabolite involved in nineteen metabolic pathways based on enrichment network analysis. In addition, histopathological analysis and body weight changes confirmed the results of the metabolomics analysis. **Conclusions**: These results provided a new perspective for the molecular mechanisms of adipose tissue toxicity induced by pentosidine.

## 1. Introduction

Pentosidine is an advanced glycation end product (AGE). It is commonly found in heat-processed foods, particularly under thermal processing at 80–100 °C. It is produced by the Maillard reaction, in which reducing sugars react with free amino groups in proteins or nucleic acids in food [1,2]. The Maillard reaction is a non-enzymatic glycation reaction, which is not only related to the sensory characteristics of food but also closely linked to human health [3]. Dietary habits have changed a lot because of urbanization and globalization. One notable phenomenon is the globally popular western-style fast food, known for its fried and baked foods, with its prevalence steadily increasing [4]. A lot of pentosidine is produced in these foods during high-temperature processing. It was reported that the pentosidine content in baked beef could reach up to 672 μg/100 g. Therefore, dietary intake has become a main source of human exposure to pentosidine [5].

It was reported that the prolonged consumption of foods rich in pentosidine may increase endogenous pentosidine levels [1], induce chronic inflammation and oxidative stress [6], and impair the function of the liver and kidneys [7]. In addition, pentosidine exposure was closely correlated with increased risks of diabetes [8], atherosclerosis [9], and other cardiovascular diseases [10]. Currently, many studies are on the formation mechanism, detection methods, and inhibition strategies of pentosidine. Grandhee and Monnier studied the formation mechanism of pentosidine in reactions involving ribose, lysine, and arginine [11]. Peiretti established an HPLC/MS method for quantifying pentosidine. It facilitated the detection of pentosidine in steaks and other food items [12]. Navarro et al. revealed that hydroxytyrosol, quercetin, gallic acid, and olive leaf extracts were able to efficiently hinder the formation of pentosidine in cookies by inhibiting Amadori product formation, an intermediate in the Maillard reaction [13].

Metabolomic technology based on mass spectrometry (MS), which detects changes in small molecules and obtains a lot of information about metabolic pathways, captures the final dynamic alteration of metabolites in reply to external stressors [14]. The technique has been used to explore the biological effects of food contaminants in organisms. Wu et al. demonstrated that a high level of AGEs in a diet hinders carbohydrate catabolism and helps lipid synthesis with metabolomics. In addition, a high level of AGEs in a diet changes gut microbiota composition and indirectly influences carbohydrate metabolism by the changing levels of glyceraldehyde and pyruvate in plasma [15]. Hu et al. used metabolomics to study potential biomarkers and metabolic pathways in the adipose tissue of rats with mepiquat exposure [16]. Adipose tissue acts as an energy reservoir for lipids, an important regulator of glucose homeostasis and an energetic endocrine organ [17]. Disrupted metabolic states are the key features of adipose tissue in response to external factors [18]. However, there are no studies on the adipose tissue of pentosidine-exposed rats by metabolomics.

In the study, non-targeted metabolomics was used to analyze the molecular mechanism of the adipose tissue toxicity of pentosidine. The aim of the study is to test the acute toxicity of pentosidine on the adipose tissue of rats. Firstly, an untargeted metabolomics approach was used to analyze the metabolic changes in adipose tissue in pentosidine-exposed rats. Differential metabolites and metabolic networks were identified. Secondly, histopathological observation and body weight changes analysis were performed to test the results of the metabolomics analysis. This study provided new insights into the molecular mechanisms of adipose tissue toxicity induced by pentosidine.

## 2. Materials and Methods

### 2.1. Chemical and Reagents

Pentosidine (purity > 98%) was obtained from Cayman Chemical (Ann Arbor, MI, USA). HPLC-grade methanol was provided by Fisher (Fair Lawn, NJ, USA). Standard compounds, 4-chloro-DL-phenylalanine, and derivatization reagent (99%MSTFA + 1%TMCS, pyridine and methoxyamine) were bought at Sigma-Aldrich (St. Louis, MO, USA). Ultra-pure water was produced by Milli-Q water system (Millipore Corp., Billerica, MA, USA). All other chemicals were analytical grade.

### 2.2. Animals and Sample Collection

Male Sprague-Dawley rats aged 5–6 weeks were kept in specific pathogen-free conditions of Department of Laboratory Animal Science of Peking University (Beijing, China). Rats were kept on 12 h light/dark cycle with unrestricted access to water and food. The environmental conditions were maintained at a temperature of 21–23 °C and a humidity of 55–65%. All experimental procedures were conducted in accordance with the European Community guidelines for experimental animal use. All animal experimental plans were agreed by Animal Care and Use Committee of Peking University (approval no. LA2019032).

Thirty rats were randomly divided into three groups of ten rats per group. Rats in the low dose (LD) group and high dose (HD) group were injected with pentosidine solution at concentrations of 0.15 mg/kg/d and 1.5 mg/kg/d via the tail vein, respectively. Rats in the normal diet (ND) group were given 0.5 mL normal saline by the same route. Rats were treated continuously for 3 days. Body weights of rats were recorded every day. On the 4th day, all rats were sacrificed. Epididymal adipose tissues were rapidly collected. Some of them were used for metabolomic analysis. The rest were kept for histopathological analysis.

### 2.3. Metabolite Extraction of Adipose Tissue

Endogenous metabolite extraction was performed by the method used in previous research [16]. The adipose tissue (0.05 g) was homogenized in 2.0 mL chloroform/methanol solution (v:v, 2:1). The supernatant was collected after centrifugation (9168× *g*, 4 °C, 5 min). The residue was extracted twice by the same procedure. The combined supernatant was centrifuged (13,201× *g*, 4 °C, 10 min) and dried with nitrogen. An amount of 15 μL 4-chloro-DL-phenylalanine (1.05 mg/mL) was added to every sample. Every sample was taken for freeze-drying for 24 h. Then, it was derivatized with MSTFA (0.1 mL) at 70 °C for 3 h [19]. After adding 170 μL chloroform, each sample was vortexed for 1.0 min and centrifuged (15,493× *g*, 4 °C, 15 min). The supernatant (0.1 mL) was used for GC-MS analysis. Quality control (QC) samples were prepared by mixing equal volumes (10 μL) of every sample. QC samples were analyzed once every six samples. They were used to monitor instrument stability and reproducibility [20].

### 2.4. GC-MS Analysis

Metabolic profiling analysis of adipose tissue was performed using an Agilent 7890A gas chromatograph linked with a 5975C mass spectrometer with an HP-5 MS capillary column (30 m × 250 μm i.d., 0.25 μm). Helium (chromatographic grade) served as the carrier gas with a stable flow rate of 1.0 mL/min. Temperature program was designed as follows: the original temperature was 80 °C and held for 2 min, raised to 180 °C at a rate of 5 °C/min, maintained for 5 min, elevated to 250 °C at a rate of 5 °C/min, held for 5 min, then raised to 280 °C at a rate of 8 ℃/min, maintained for 2 min. The injector temperature was 280 °C. Auxiliary heating was set at 280 °C. Injection volume was 1 μL. Solvent delay was 5 min. Splitless injection mode was used in analysis. Electron energy was 70 eV. Mass data were obtained in full scan mode covering a mass range of 50–650 *m*/*z*. Compound identification was finished by standards and the NIST database (2014) (Gaithersburg, MD, USA).

### 2.5. Statistical Analysis

Metabolomics data analysis was finished based on previous research [16]. Normalized data were input into SIMCA 14.1 software (Umetrics, Umeå, Sweden) and analyzed by partial least-squares discriminant analysis (PLS-DA) and principal components analysis (PCA) models. Mann–Whitney U test from SPSS 22.0 (SPSS Inc., Chicago, IL, USA) provided *p*-values. Differential metabolites were screened according to variable importance in projection (VIP) (VIP > 1) and *p*-values (*p* < 0.05). Boxplots were obtained by Origin2021 (OriginLab, Northampton, MA, USA). Metabolic pathway analysis was completed by Metaboanalyst 5.0 website (https://www.metaboanalyst.ca/ (accessed on 2 August 2023)) and Cytoscape v3.7.2 software. Kyoto Encyclopedia of Genes and Genomes (KEGG) and Adobe Illustrator 2023 software were used for analyzing the disrupted metabolic pathways in rats with pentosidine exposure.

### 2.6. Receiver Operating Characteristic (ROC) Curve Analysis

The area under the ROC curve (AUC) was calculated using SPSS 22.0 software. It was used to assess the ability of differential metabolites to distinguish pentosidine exposure groups from normal diet group. AUC values from 0.5 to 1.0 showed discrimination accuracy. AUC values above 0.8 revealed high discriminatory ability of differential metabolites.

### 2.7. Histopathological Analysis

Adipose tissues of rats were kept in 10% neutral formalin and processed for paraffin embedding. Samples 5 μm thick were cut and mounted on slides. These slices were stained by hematoxylin and eosin (H&E) for histological analysis.

## 3. Results and Discussion

### 3.1. Weight Change

**Figure 1:**
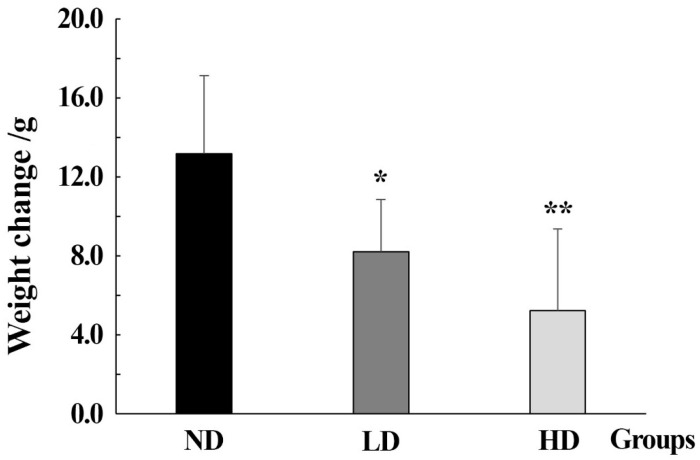
Weight changes in rats in ND, LD, and HD groups. Data were expressed as mean ± standard deviation (SD). Differences in different groups were evaluated by *t*-test, “*” represents *p* < 0.05, “**” represents *p* < 0.01. ND group: normal diet group, n = 10; LD group: low dose group, n = 10; HD group: high dose group, n = 10.

Compared to the ND group, the body weight changes decreased in rats from the pentosidine-exposed groups. It showed a dose-dependent effect. It suggested that pentosidine significantly inhibited the body weight gain of the rats (*p* < 0.05), as shown in Figure 1.

### 3.2. Histopathological Analysis

**Figure 2:**
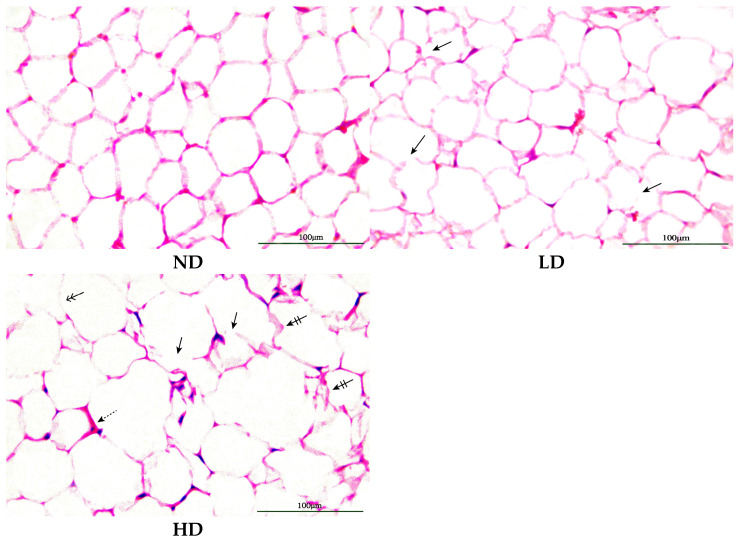
H&E staining images of adipose tissue from ND, LD, and HD groups (original magnification: 400×). Black arrows: damaged cell membranes; barred arrows: distorted cell contours; doubleheaded arrows: blurred cell contours; dashed arrows: inflammatory cell. ND: normal diet group; LD: low dose group; HD: high dose group.

Adipose tissue samples of rats were analyzed histopathologically. As shown in Figure 2, the adipocytes in the ND group were arranged neatly and closely, with regular size and clear cell contours. It exhibited different sizes and irregular alterations in cells in the LD group. Cell membranes appeared to collapse. In the HD group, there were remarkable changes in the size and shape in the adipocytes, with some cells significantly larger than those in the ND group. Additionally, the contours of cells in the HD group were distorted and blurred. It showed damaged cell membranes. Inflammatory cell infiltration was found. Based on histological alternations, it can be concluded that pentosidine exhibited toxicity in the adipose tissue of rats. The study was to comprehend the pentosidine effects on adipose tissue at the molecular level. Therefore, metabolomics was conducted to investigate how pentosidine influenced the adipose tissue.

### 3.3. GC-MS Analysis of Adipose Tissue

Differential metabolites were screened based on VIP > 1 from the multivariate analysis and *p* < 0.05 from the Mann–Whitney U test. A total of forty-two differential metabolites in the adipose tissue were quantified by normalization to internal standard (4-chloro-DL-phenylalanine). The relative levels of metabolites were presented as fold changes (FC). Compared to the ND group, sixteen upregulated metabolites were identified in the HD group, including glycine, pyrimidine, L-threonine, L-homoserine, 2,5-cyclohexadiene-1,4-dione, anthracene, acetic acid, glutamine, pyruvic acid, D-ribose, iron, glutaconic acid, cis-9-hexadecenoic acid, hexadecanoic acid, inositol, and eicosapentaenoic acid, while twenty-six downregulated metabolites were identified in the HD group, including ribitol, L-leucine, L-serine, cyclohexanecarboxylic acid, fumaric acid, α-ketoglutaric acid, 2-oxovaleric acid, malonic acid, L-methionine, heptadecane, 2-ketoisocaproic acid, D-glucuronic acid, propionic acid, estrone, sulfurous acid, caprylic acid, naphthalene, butyric acid, benzoic acid, 1,2-benzenedicarboxylic acid, L-ascorbic acid, octadecanoic acid, petroselinic acid, myristic acid, cholesterol, and adipic acid (Table 1). These differential metabolites mainly belong to amino acids, fatty acids, carbohydrates, carboxylic acids, steroids, ketones and their derivatives, as well as benzoic acid and its derivatives, with fatty acids being the most prevalent, followed by amino acids, carboxylic acids, and carbohydrates. In short, our findings indicated that pentosidine acute exposure substantially changed the levels of metabolites in adipose tissue.

### 3.4. Metabolomics Analysis

**Figure 3:**
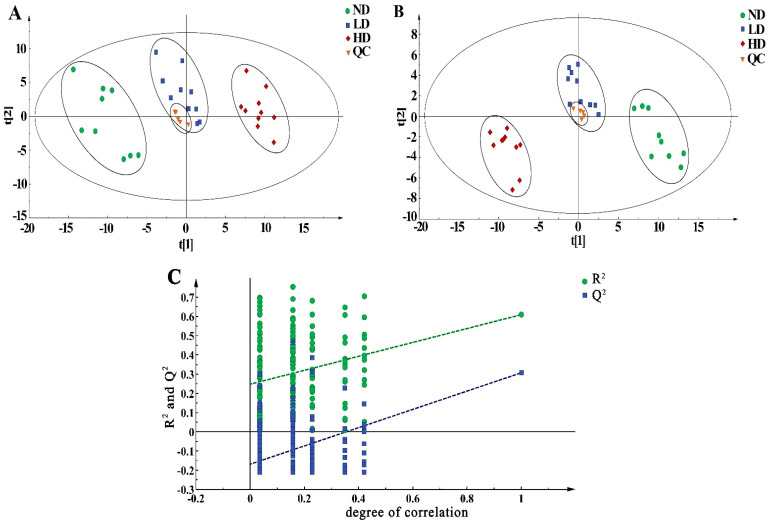
Multivariate statistical analysis of results from GC−MS analysis. (**A**) PCA score plot analysis (R^2^X =74.2%, Q^2^ = 49.9%); (**B**) PLS−DA score plot analysis (R^2^X = 81.9%, R^2^Y = 99.4%, Q^2^ = 92.8%); (**C**) permutation plot for PLS−DA model (n = 999), R^2^ = (0.0, 0.253), Q^2^ = (0.0, − 0.177). PCA: principal component analysis; PLS−DA: partial least squares discriminant analysis; ND: normal diet group, n = 10; LD: low dose group, n = 10; HD: high dose group, n = 10; QC: quality control.

PCA and PLS-DA score plots are shown in Figure 3. The close aggregation of QC samples in PCA and PLS-DA models revealed the reliability and stability of metabolomics data. In Figure 3A, it shows that samples of the ND, LD, and HD groups were significantly separated. The parameters were R^2^X = 74.2% and Q^2^ = 49.9%, indicating the good quality and predictive ability of the PCA model. It suggested that pentosidine exposure has a remarkable impact on metabolic profiles in adipose tissue.

PLS-DA model was established to assess the metabolic patterns altered by pentosidine. It can better distinguish differences among the three groups. The 999-time permutation tests were utilized to verify the accuracy and predictive ability of the model. As depicted in Figure 3C, the permuted R^2^Y value was <0.4, and the Q^2^Y value was <0.05. Therefore, the permutation test indicated that the PLS-DA model was not overfitted. Clear separation among the ND, LD, and HD groups and great parameters (R^2^X = 81.9%, R^2^Y = 99.4%, Q^2^ = 92.8%) of the PLS-DA model suggest remarkable metabolic changes induced by pentosidine in a dose-dependent manner (Figure 3B).

**Figure 4:**
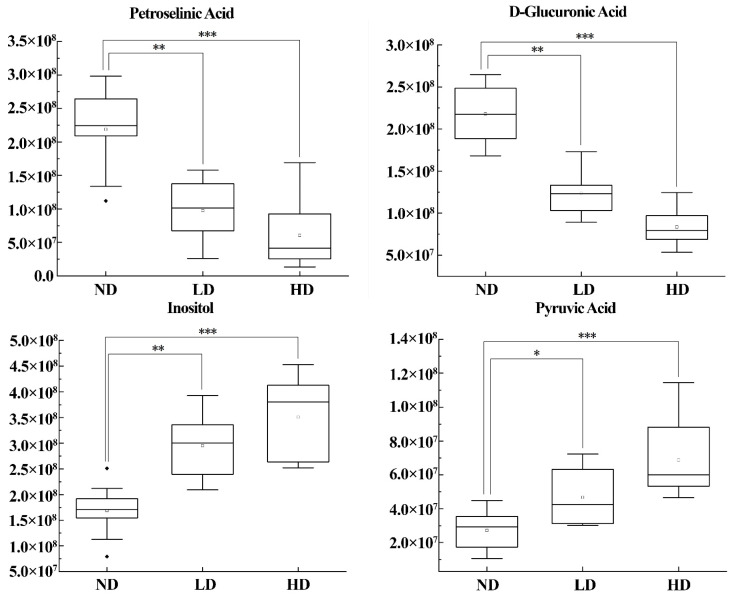
Boxplots of differential metabolites in adipose tissue of rats in ND, LD, and HD groups. Differences in different groups were evaluated by Mann–Whitney U test. “*” means *p* < 0.05, “**” means *p* < 0.01, “***” means *p* < 0.001. ND group: normal diet group, n = 10; LD group: low dose group, n = 10; HD group: high dose group, n = 10.

Boxplots were used to show changes in the relative levels of differential metabolites in the HD, LD, and ND groups (Figure 4). Compared to the ND group, the levels of inositol and pyruvic acid increased in the pentosidine-exposed groups. In contrast, the amounts of petroselinic acid and D-glucuronic acid content decreased in the pentosidine-exposed groups. These changes showed a dose-dependent relationship in the three groups.

### 3.5. ROC Curve

The ability of these differential metabolites to distinguish the normal diet group from the pentosidine-exposed groups was assessed by AUC values. Each differential metabolite was characterized by the AUC value ranging from 0.8 and 1. Thus, forty-two differential metabolites can effectively distinguish rats in the ND group from those in the pentosidine-exposed groups (Table S1).

### 3.6. Metabolic Pathway Analysis

**Figure 5:**
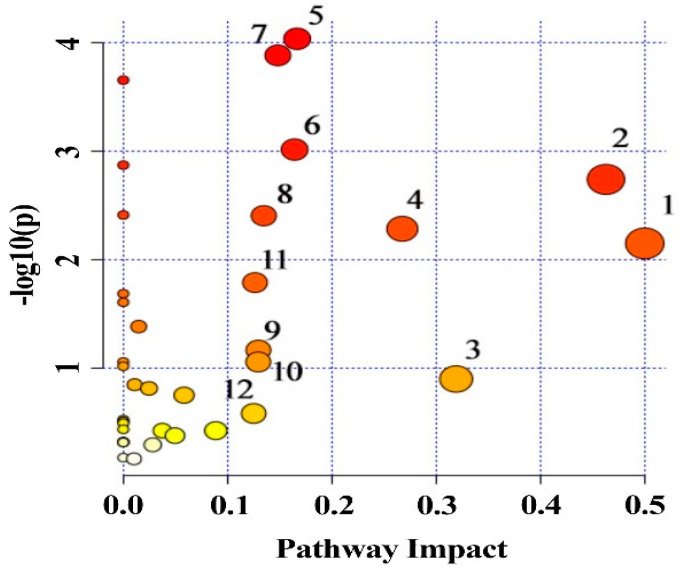
Disturbed pathways in adipose tissue of rats from ND, LD, and HD groups. Node color in pathway analysis represented its *p*-value; node radius reflected their pathway impact values. (1) Ascorbate and aldarate metabolism, (2) glycine, serine, and threonine metabolism, (3) sulfur metabolism, (4) pyruvate metabolism, (5) aminoacyl−tRNA biosynthesis, (6) alanine, aspartate, and glutamate metabolism, (7) glyoxylate and dicarboxylate metabolism, (8) citrate cycle (TCA cycle), (9) glycolysis/gluconeogenesis, (10) inositol phosphate metabolism, (11) cysteine and methionine metabolism, (12) pentose and glucuronate interconversions. ND group: normal diet group, n = 10; LD group: low dose group, n = 10; HD group: high dose group, n = 10.

Forty-two differential metabolites were input into the MetaboAnalyst 5.0 website for pathway analysis. It linked changes in metabolites to metabolic networks. There were twenty-one metabolic pathways enriched in the adipose tissue. Twelve of them were perturbed significantly (the impact threshold sourced from the pathway analysis is above 0.1), such as ascorbate and aldarate metabolism, glycine, serine, and threonine metabolism, sulfur metabolism, pyruvate metabolism, aminoacyl-tRNA biosynthesis, alanine, aspartate and glutamate metabolism, glyoxylate and dicarboxylate metabolism, citrate cycle (TCA cycle), glycolysis/gluconeogenesis, inositol phosphate metabolism, cysteine and methionine metabolism, and pentose and glucuronate interconversions (Figure 5 and Table S2). The results exhibited disruptions in carbohydrate metabolism, energy metabolism, amino acid metabolism, and the other metabolic pathways of adipose tissue induced by pentosidine. The most remarkably impacted pathway was ascorbate and aldarate metabolism (Figure S1), a significant carbohydrate metabolic pathway that prevents cells from oxidative damage [21]. In short, this indicated the possible impacts of pentosidine acute exposure on metabolic pathways.

**Figure 6:**
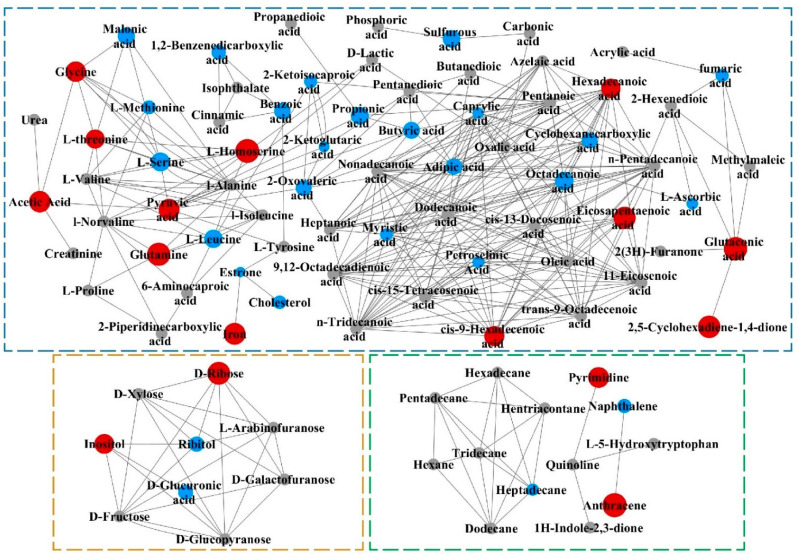
Pathway mapping of differential metabolites detected in HD group compared to ND group. Metabolic pathway generated through MetaMapp and drawn by Cytoscape. The depicted network reveals that red nodes represent significantly upregulated metabolites, blue nodes show remarkably downregulated metabolites, and gray nodes reveal no significant changes in metabolites. Size of node is positively correlated with fold change between HD group and ND group. ND group: normal diet group, n = 10; LD group: low dose group, n = 10; HD group: high dose group.

Based on the chemical structures of metabolites and the functional groups classification from PubChem, MetaMapp was used to form the metabolic network related to differential metabolites. The molecular metabolite network was constructed to obtain an overall view of metabolomics datasets in Cytoscape software (Figure 6). The blue-dotted box revealed amino acid, energy, and fatty acid metabolism. The yellow-dotted box showed carbohydrate metabolism. The green-dotted box was related to nucleotide metabolism and other pathways. The comprehensive metabolic pathway of metabolites in adipose tissue accurately reflected its metabolic state. There were both upregulated and downregulated metabolites among the forty-two differential metabolites. L-Homoserine showed the largest change among upregulated metabolites, while L-serine had the greatest change among downregulated metabolites. It indicated that pathways linked to L-homoserine and L-serine were particularly affected, such as glycine, serine, and threonine metabolism and cysteine and methionine metabolism. It highlighted the influence of pentosidine on amino acid metabolism in adipose tissue. Notably, a series of fatty acids and their derivatives, carbohydrates, carboxylic acids, and steroids displayed significant changes. Pentosidine caused more remarkable disruption in fatty acid metabolism.

**Figure 7:**
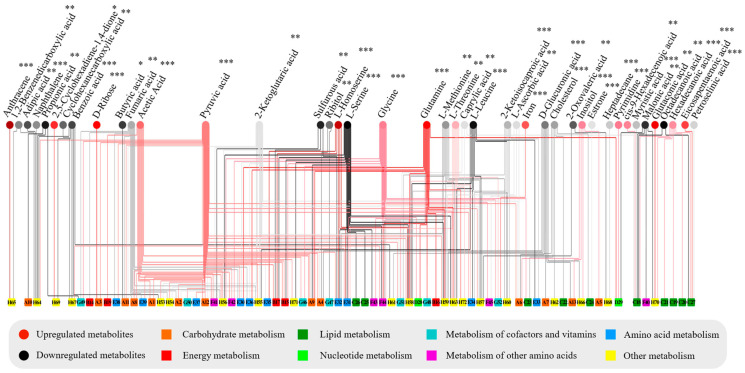
Network analysis of differential metabolites and metabolic pathways in pentosidine-exposed groups shows that there are forty-two differential metabolites. “*” represents *p* < 0.05, “**” represents *p* < 0.01, “***” represents *p* < 0.001. The red circles and black circles show upregulated and downregulated metabolites, respectively. Intensity of colors indicates fold changes in metabolites. A total of seventy-two metabolic pathways are classified as eight metabolic pathways (Table S3) and connected to different metabolites by the red line (upregulated) and black line (downregulated).

**Figure 8:**
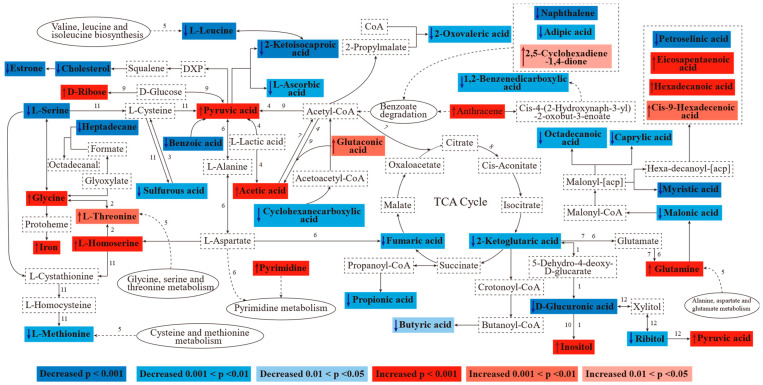
Interplay between differential metabolites in HD group compared to ND group. Metabolic pathways are illustrated based on information obtained from KEGG database. Red boxes represent increased metabolites, blue boxes show decreased metabolites, blank boxes reveal no significant changes in metabolites. Numbers 1 to 12 represent metabolic pathways with impact value larger than 0.1 (Figure 5). ND group: normal diet group, n = 10; LD group: low dose group, n = 10; HD group: high dose group, n = 10.

As shown in Figure 7, twenty-six downregulated and sixteen upregulated metabolites were significantly altered by pentosidine in adipose tissue. Seventy-two metabolic pathways involving eight routes were impacted (Table S3). Amino acid, carbohydrate, and fatty acid metabolisms were notably affected, especially with downregulation in unsaturated fatty acid biosynthesis and steroid biosynthesis. The dysregulation of pyruvic acid and α-ketoglutaric acid affected thirty-four and nineteen pathways, respectively. Therefore, pyruvic acid was regarded as a possible key upregulated metabolite. α-Ketoglutaric acid was considered as a probable key downregulated metabolite. Based on the KEGG database, an overview of significantly changed metabolites and pathways in the adipose tissue of rats with pentosidine exposure is shown in Figure 8.

### 3.7. Metabolic Disturbance of Adipose Tissue after Pentosidine Exposure

Amino acid metabolism plays an essential role in cellular function, such as cellular signaling and protein and lipid synthesis. One of the most remarkably changed pathways between the pentosidine-exposed groups and the ND group was glycine, serine, and threonine metabolism (Figure 5) in this study. It provides nicotinamide adenine dinucleotide phosphate (NADPH) and participates in the TCA cycle, gluconeogenesis, and macromolecular biosynthesis [22]. Serine, glycine, and threonine can be converted to each other by the action of threonine aldolase and serine hydroxymethyltransferase [23]. Glycine serves as an effective nitrogen source and can be converted into pyruvate, subsequently entering the TCA cycle as an amino acid skeleton. It plays crucial roles in metabolic regulation, neurological function, and antioxidant response [24]. It has been shown that L-threonine is required for the inhibition of lipogenesis and the promotion of lipolysis in epididymal adipose tissue [25]. One-carbon units produced by L-serine are crucial for heat generation and ATP in adipose cells [26]. L-serine is a key component in the synthesis of phospholipids, especially phosphatidylserine and sphingolipids, which are highly concentrated in all cellular membranes [27]. Homoserine is a more reactive variant of serine. It is also involved in the metabolism of glycine, serine, and threonine [24]. Therefore, we believed that a significant increase in glycine, L-threonine, and L-homoserine, along with a notable decrease in L-serine, indicated the disruption in glycine, serine, and threonine metabolism, which may result in adipose tissue disorders. Glutamine, a vital non-essential amino acid, is abundant in the human body. High levels of glutamine can help cancer cell proliferation by acting as carbon and nitrogen sources for fatty acids, nucleotides, and non-essential amino acids synthesis. Glutamine is also included in glutathione and NADPH production. Additionally, it is a potent anaplerotic substrate to maintain the TCA cycle [28,29]. Therefore, the significant increase in glutamine revealed disturbances in glutamate and glutamine pathways. This possibly impacted the TCA cycle. It can be found in a remarkable decrease in α-ketoglutaric acid, an intermediate product of the TCA cycle (Figure 8). Pentosidine exposure also resulted in a significant decrease in L-leucine and 2-ketoisocaproic acid, along with a remarkable increase in glutaconic acid. An intermediate of leucine metabolism, 2-Ketoisocaproic acid, might enter the cellular energy metabolism by 2-keto acid dehydrogenase [30]. Leucine also acts as a regulator of lipid metabolism, helping to reduce obesity and fat accumulation [31]. These changes indicated disorders of energy metabolism and lipid metabolism in adipose tissue. An increase in reactive oxygen species (ROS) in cells in an inflammatory environment will induce the oxidation of sulfur in L-methionine residues, forming L-methionine-S-oxide. It will lower the level of L-methionine. The lack of L-methionine will result in a deficient supply of sulfur in cells. Sulfur is crucial for the formation of iron–sulfur clusters in cells. These clusters can decrease iron accumulation to prevent the occurrence of ferroptosis [32,33]. Additionally, benzoic acid is naturally found in animal tissues. It shows a negative correlation with ROS [34,35]. Therefore, the downregulation of benzoic acid in pentosidine-exposed groups might induce oxidative stress. A decrease in L-methionine and an increase in iron after pentosidine acute exposure suggested that L-methionine may be oxidized to become L-methionine-S-oxide. Pyrimidine is a fundamental constituent of DNA and RNA. Pyrimidine metabolic abnormality disturbed nervous, hematological, or mitochondrial systems [36,37]. Additionally, 2,5-cyclohexadiene-1,4-dione is known to be involved in oxidative stress and DNA damage [38]. The increase in 2,5-cyclohexadiene-1,4-dione in this study indicated its potential harm.

The increase in pyruvic acid, along with the decrease in fumaric acid and α-ketoglutaric acid, was observed (Figure 6) in the pentosidine-exposed groups. They were involved in pyruvate metabolism and the TCA cycle, respectively. The TCA cycle, as the central hub of cellular energy metabolism, coordinates metabolisms of glucose, fatty acids, and amino acids. Metabolites of the TCA cycle also take part in nonenergy production activities, including biosynthesis, maintaining redox homeostasis, and modulating inflammation [39]. Pyruvic acid is a key compound in energy metabolism. It is decarboxylated with pyruvate dehydrogenase complex. It gives an additional NADPH molecule and acetyl-coenzyme A, an entry-level substrate for the TCA cycle [40]. Significant alterations in fumaric acid, α-ketoglutaric acid, and pyruvic acid indicated a negative influence of pentosidine acute exposure on interconnected pathways such as pyruvate metabolism and the TCA cycle. Ribitol, as a reduction product of D-ribose, enhances glycolysis by increasing the production of pyruvic acid and lactic acid [41]. Compared to the ND group, an increase in D-ribose and a decrease in ribitol in pentosidine-exposed groups might disturb ribose metabolism and glycolysis/gluconeogenesis pathways, potentially leading to inflammatory responses [42]. As a precursor of lipids and inositol phosphates, inositol plays a significant biological role in growth regulation, signaling, and membrane lipid synthesis. Moreover, inositol can be converted to D-glucuronic acid and enter the pentose phosphate pathway [43]. D-Glucuronic acid is considered the most powerful detoxifying natural compound in an organism due to its ability to conjugate itself with noxious metabolites or waste products [44]. Additionally, as a component of hyaluronic acid, D-glucuronic acid is implicated in the regulation of the extracellular matrix and inflammatory conditions in adipose tissue [45]. L-Ascorbic acid was shown to inhibit free radical generation and reduce oxidative damage resulting from ROS [46]. Therefore, significant changes in inositol, D-glucuronic acid, and L-ascorbic acid indicated disruptions in inositol phosphate metabolism, pentose and glucuronate interconversions, and ascorbate and aldarate metabolism, revealing the dysfunction of adipose tissue.

In addition to being included in energy metabolism, short-chain fatty acids (SCFAs) can activate G-protein-coupled receptors as signaling molecules [47]. In this study, there was a notable rise in acetic acid, while levels of propionic acid and butyric acid decreased significantly. Propionic acid and butyric acid reduced the total fat mass, subcutaneous fat mass, and macrovesicular steatosis [48,49]. Butyric acid can also help brown–white tissue, decrease the size of adipose cells in morphology, and increase the number of multicellular adipocytes [50]. Notable alterations in the levels of SCFAs may result in fatty acid β-oxidation, inflammation, and insulin resistance [51]. Sulfurous acid is formed by the catabolism of cysteine and methionine. It improves intracellular glutathione to keep neurons from oxidative stress [52]. As shown in Figure 5, the cysteine and methionine metabolism were significantly disturbed in the adipose tissue of rats with pentosidine acute exposure. Therefore, we believed that a decrease in sulfurous acid was in connection with the disruption of cysteine and methionine metabolism. It might lead to imbalances in glutathione biosynthesis and oxidative stress.

The metabolites associated with fatty acid metabolism, such as malonic acid, caprylic acid, octadecanoic acid, and so on, exhibited significant changes in this study. White adipose tissue was widely considered an energy homeostasis center for the lipolysis of fatty acids (FAs) and the lipid storage of the whole body. FAs have many fates including oxidation to generate ATP for energy, reesterification back into triacylglycerols, and also serving as signaling molecules [53]. FAs are also important parts for membrane structure lipids [17]. In addition, excessive FAs released by the adipocyte are regarded as a major contributor to tissue inflammation and can lead to various metabolic diseases [54]. Therefore, changes in FAs indicated a dysregulation in fatty acid metabolism in the adipose tissue of rats with pentosidine acute exposure. Malonic acid plays an important role in fatty acid biosynthesis under the action of malonyl-CoA. Malonyl-CoA is an important intermediate metabolite in fatty acid biosynthesis. It is reversibly changed to malonic acid in cells [55]. Therefore, a decrease in malonic acid caused by pentosidine acute exposure suggested a disruption of fatty acid biosynthesis in the adipose tissue. Since caprylic acid is ketogenic, it could offer an additional source of energy, generally in the form of β-hydroxybutyrate, which aborts impaired glucose metabolism in Alzheimer’s disease [56]. Additionally, caprylic acid induced a large reduction in the expression of crucial enzymes in FA uptake and triglyceride synthesis [57]. Therefore, a decrease in caprylic acid might promote triglyceride production, the disorder of lipid metabolism, and insufficient energy supply for patients with Alzheimer’s disease. Compared to other saturated fatty acids, octadecanoic acid inhibits cell growth at specific concentrations [58]. In addition, it is also a regulator of mitochondrial function. The loss of octadecanoic acid leads to mitochondrial fragmentation, which will accelerate the production of ROS and apoptosis [59]. Thus, a decrease in octadecanoic acid in this study might seriously interfere with the normal function of adipocytes. It might also increase the production of triglyceride substrates, such as cis-9-hexadecenoic acid. High contents of cis-9-hexadecenoic acid quicken fat deposition [60]. Furthermore, pentosidine helped the accumulation of saturated fatty acids, including hexadecanoic acid. It was reported that saturated fatty acids were the major contributors for lipotoxicity by helping oxidative stress and cell apoptosis [61]. Myristic acid performed an anti-inflammatory effect by increasing IL-10. Myristic acid slightly modulated the activity of peroxisome proliferator activated receptor α, a nuclear receptor with a crucial role in the regulation of mitochondrial metabolism and mitochondrial biogenesis [62]. Therefore, a decrease in myristic acid in this study could hinder its vital physiological function in adipose tissue. Petroselinic acid helped improve synthesis and the collection of eicosapentaenoic acid (EPA). EPA stabilized membranes and maintained a normal distribution of membrane cholesterol [63]. It was found that EPA helped ABCA1-mediated cholesterol efflux from macrophages. It was the main mechanism to remove cholesterol from tissues to the liver [64]. These alterations were correlated with an increase in EPA. In this study, changes in petroselinic acid and EPA were consistent with the literature [63]. Cholesterol is a primary components of the cell membrane. It participates in the regulation of permeability, fluidity of the membrane, and cell signaling [65]. Thus, a decrease in cholesterol and estrone in the study could disrupt cholesterol metabolism and steroid hormone synthesis (Figure 7).

## 4. Conclusions

It is the first time that an untargeted metabolomics study has been completed to investigate the possible toxic mechanism of pentosidine on adipose tissue. Forty-two differential metabolites were identified. Sixteen metabolites were significantly upregulated. Conversely, twenty-six metabolites were significantly downregulated. The most important upregulated metabolite was possibly pyruvic acid, which was involved in thirty-four metabolic pathways. α-Ketoglutaric acid was a probable key downregulated metabolite involved in nineteen metabolic pathways. Major disturbed pathways, including ascorbate and aldarate metabolism, glycine, serine, and threonine metabolism, sulfur metabolism, pyruvate metabolism, aminoacyl-tRNA biosynthesis, alanine, aspartate, and glutamate metabolism, glyoxylate and dicarboxylate metabolism, TCA cycle, glycolysis/gluconeogenesis, inositol phosphate metabolism, cysteine and methionine metabolism, and pentose and glucuronate interconversions were found based on the pathway analysis. Changes in the levels of amino acids, fatty acids, carbohydrates, and other metabolites and the disturbance of energy metabolism, fatty acid, and amino acid metabolism were found in this study. In addition, the histopathological analysis of adipose tissue and body weight changes confirmed the results of the metabolomics analysis. It may provide a new perspective on the molecular mechanisms of adipose toxicity induced by pentosidine.

## Figures and Tables

**Table 1:** Differential metabolites detected in high dose group compared to normal diet group by metabolomic analysis based on GC-MS.

No.	Metabolite	RT ^1^ (min)	*p*-Value	VIP	FC ^2^ (HD-ND)	FDR ^3^	Trend ^4^	Class ^5^	Metabolic Pathways
1	Glycine	7.4	2.39 × 10^−4^	1.06	2.10	1.0 × 10^−2^	↑	Amino acids, peptides, and analogues	Glycine, serine, and threonine metabolism
2	Ribitol	10.4	3.28 × 10^−3^	1.08	0.40	4.59 × 10^−3^	↓	Carbohydrates and carbohydrate conjugates	Pentose and glucuronate interconversions
3	L-Leucine	11.1	8.15 × 10^−4^	1.02	0.56	1.71 × 10^−3^	↓	Amino acids, peptides, and analogues	Valine, leucine, and isoleucine biosynthesis
4	Pyrimidine	12.5	2.39 × 10^−4^	1.18	2.51	5.02 × 10^−3^	↑	Pyrimidines and pyrimidine derivatives	Pyrimidine metabolism
5	L-Serine	13.4	6.05 × 10^−4^	1.03	0.60	1.69 × 10^−3^	↓	Amino acids, peptides, and analogues	Glycine, serine, and threonine metabolism
6	Cyclohexanecarboxylic acid	13.5	4.28 × 10^−3^	1.06	0.46	5.44 × 10^−3^	↓	Carboxylic acids	Benzoate degradation
7	Fumaric acid	13.9	1.08 × 10^−2^	1.24	0.47	1.14 × 10^−2^	↓	Dicarboxylic acids and derivatives	TCA cycle
8	L-Threonine	14.1	3.29 × 10^−3^	1.05	1.66	4.46 × 10^−3^	↑	Amino acids, peptides, and analogues	Glycine, serine, and threonine metabolism
9	α-Ketoglutaric acid	14.2	1.19 × 10^−3^	1.10	0.24	2.28 × 10^−3^	↓	Gamma-keto acids and derivatives	TCA cycle
10	2-Oxovaleric acid	14.9	7.37 × 10^−3^	1.05	0.47	8.37 × 10^−3^	↓	Short-chain keto acids and derivatives	Lipid metabolism
11	L-Homoserine	15.5	6.36 × 10^−4^	1.21	5.58	1.57 × 10^−3^	↑	Amino acids, peptides, and analogues	Glycine, serine, and threonine metabolism
12	2,5-Cyclohexadiene-1,4-dione	16.0	2.01 × 10^−2^	1.23	3.21	2.06 × 10^−2^	↑	Carbonyl compounds	Aminobenz oate degradation
13	Malonic acid	16.1	1.38 × 10^−3^	1.04	0.53	2.32 × 10^−3^	↓	Dicarboxylic acids and derivatives	Fatty acid metabolism
14	L-Methionine	16.2	1.75 × 10^−3^	1.12	0.38	2.82 × 10^−3^	↓	Amino acids, peptides, and analogues	Cysteine and methionine metabolism
15	Heptadecane	16.6	3.79 × 10^−4^	1.11	0.29	1.22 × 10^−3^	↓	Alkanes	Lipid metabolism
16	2-Ketoisocaproic acid	16.9	2.39 × 10^−4^	1.01	0.33	3.35 × 10^−3^	↓	Short-chain keto acids and derivatives	Valine, leucine, and isoleucine biosynthesis
17	D-Glucuronic acid	17.5	3.79 × 10^−4^	1.03	0.38	1.14 × 10^−3^	↓	Carbohydrates and carbohydrate conjugates	Amino sugar and nucleotide sugar metabolism
18	Propionic acid	17.9	5.50 × 10^−3^	1.03	0.54	6.60 × 10^−3^	↓	Carboxylic acids	Propanoate metabolism
19	Anthracene	18.8	6.36 × 10^−4^	1.35	7.92	1.48 × 10^−3^	↑	Anthracenes	Polycyclic aromatic hydrocarbon degradation
20	Acetic acid	18.9	7.69 × 10^−3^	1.09	1.90	8.50 × 10^−3^	↑	Carboxylic acids and derivatives	Glycolysis/gluconeogenesis
21	Estrone	19.3	8.48 × 10^−3^	1.09	0.12	1.70 × 10^−3^	↓	Estrone steroids	Steroid hormone biosynthesis
22	Glutamine	19.7	6.36 × 10^−4^	1.07	3.93	4.41 × 10^−3^	↑	Amino acids, peptides, and analogues	Alanine, aspartate, and glutamate metabolism
23	Pyruvic acid	20.0	2.39 × 10^−4^	1.03	2.54	2.51 × 10^−3^	↑	Alpha-keto acids and derivatives	TCA cycle
24	D-Ribose	20.6	2.39 × 10^−4^	1.31	3.59	2.01 × 10^−3^	↑	Carbohydrates and carbohydrate conjugates	Pentose phosphate pathway
25	Iron	21.1	2.39 × 10^−4^	1.07	2.86	1.67 × 10^−3^	↑	Homogeneous transition metal compounds	Porphyrin metabolism
26	Sulfurous acid	21.3	1.79 × 10^−3^	1.01	0.54	2.79 × 10^−3^	↓	Non-metal sulfites	Cysteine and methionine metabolism
27	Caprylic acid	21.8	2.66 × 10^−3^	1.01	0.26	3.86 × 10^−3^	↓	Fatty acids and conjugates	Fatty acid biosynthesis
28	Naphthalene	22.1	3.49 × 10^−4^	1.08	0.38	1.33 × 10^−3^	↓	Naphthalenes	Degradation of aromatic compounds
29	Butyric acid	22.8	1.06 × 10^−2^	1.26	0.53	1.14 × 10^−2^	↓	Fatty acids and conjugates	Butanoate metabolism
30	Glutaconic acid	22.9	1.19 × 10^−3^	1.43	5.134	2.18 × 10^−3^	↑	Dicarboxylic acids and derivatives	Fatty acid metabolism
31	Benzoic acid	27.3	2.39 × 10^−4^	1.07	0.50	1.43 × 10^−3^	↓	Benzoic acids and derivatives	Benzoate degradation
32	1,2-Benzenedicarboxylic acid	28.2	3.37 × 10^−3^	2.24	0.38	4.42 × 10^−3^	↓	Benzoic acids and derivatives	Polycyclic aromatic hydrocarbon degradation
33	L-Ascorbic acid	29.9	5.48 × 10^−3^	1.82	0.26	6.77 × 10^−3^	↓	Furanones	Ascorbate and aldarate metabolism
34	Cis-9-Hexadecenoic acid	30.6	1.37 × 10^−3^	1.27	2.09	2.40 × 10^−3^	↑	Fatty acids and conjugates	Fatty acid biosynthesis
35	Hexadecanoic acid	31.6	4.12 × 10^−2^	1.05	1.27	4.12 × 10^−2^	↑	Fatty acids and conjugates	Fatty acid biosynthesis
36	Inositol	33.0	3.49 × 10^−4^	1.03	2.08	1.22 × 10^−3^	↑	Alcohols and polyols	Inositol phosphate metabolism
37	Octadecanoic acid	35.6	1.92 × 10^−3^	1.04	0.56	2.88 × 10^−3^	↓	Fatty acids and conjugates	Biosynthesis of unsaturated fatty acids
38	Eicosapentaenoic acid	38.3	2.39 × 10^−3^	1.31	3.02	1.25 × 10^−3^	↑	Fatty acids and conjugates	Biosynthesis of unsaturated fatty acids
39	Petroselinic acid	38.8	6.05 × 10^−4^	1.10	0.28	1.59 × 10^−3^	↓	Fatty acids and conjugates	Biosynthesis of unsaturated fatty acids
40	Myristic acid	39.0	2.39 × 10^−4^	1.25	0.32	1.11 × 10^−3^	↓	Fatty acids and conjugates	Fatty acid biosynthesis
41	Cholesterol	42.0	2.39 × 10^−4^	1.07	0.34	1.00 × 10^−3^	↓	Cholestane steroids	Cholesterol metabolism
42	Adipic acid	42.8	7.05 × 10^−3^	1.04	0.50	8.23 × 10^−3^	↓	Fatty acids and conjugates	Degradation of aromatic compounds

^1^ RT: retention time. ^2^ FC: fold change. It represents the ratio of peak intensity of high dose group to normal diet group. ^3^ FDR: false discovery rate. ^4^ Trend: “↑” represents an increase in differential metabolites in high dose group compared to normal diet group; “↓” means a decrease in differential metabolites in high dose group compared to normal diet group. ^5^ Class: obtained from Human Metabolome Database (HMDB).

## Data Availability

All data are within the manuscript and Supplementary Materials.

## Supplementary Materials

The following supporting information can be downloaded at: https://www.mdpi.com/article/10.3390/metabo14100539/s1, Figure S1: Ascorbate and aldarate metabolic pathway in adipose tissue of rats among different groups; Table S1: Parameters of the receiver operating characteristic curves for differential metabolites; Table S2: Pathway analysis results of differential metabolites with MetaboAnalyst 5.0; Table S3: The list of metabolic pathways.
