# Supplementary material for: Untargeted Metabolite Profiling Reveals Acute Toxicity of Pentosidine on Adipose Tissue of Rats
Source: Metabolites. 2024 Oct 9;14(10):539. doi: 10.3390/metabo14100539 (PMC11509468; doi:10.3390/metabo14100539)
Supplement: Supplementary file 1 [file metabolites-14-00539-s001.zip › metabolites-3202605-supplementary.pdf]

Supplementary File

Untargeted Metabolite Profiling Reveals Acute Toxicity of Pentosidine on Adipose Tissue of Rats

Chuanqin Hu [1], Zhenzhen Shao [1], Wei Wu [1] and Jing Wang [2,*]

1 School of Light Industry Science and Engineering, Beijing Technology and Business University (BTBU), 11 Fucheng Road, Beijing 100048, China; huchuanqin@btbu.edu.cn (C.H.)
2 Key Laboratory of Geriatric Nutrition and Health, Beijing Technology and Business University, Ministry of Education, Beijing 100048, China
* Correspondence: wangjing@th.btbu.edu.cn; Tel.: +86-010-68985334; Fax: +86-010-68985378

Table S1. Parameters of the receiver operating characteristic curves for differential metabolites.

Metabolite	AUC	Standard error	Sig.
Glycine	0.8778	0.080	0.006
Ribitol	0.9375	0.059	0.003
L-Leucine	0.9555	0.043	0.001
Pyrimidine	0.9333	0.056	0.001
L-Serine	0.9667	0.035	0.001
Cyclohexanecarboxylic acid	0.9762	0.037	0.004
Fumaric acid	0.8750	0.106	0.020
L-Threonine	0.9300	0.056	0.001
α-Ketoglutaric acid	0.9286	0.066	0.005
2-Oxovaleric acid	0.9714	0.044	0.007
L-Homoserine	0.8571	0.092	0.015
2,5-Cyclohexadiene-1,4-dione	0.9444	0.080	0.039
Malonic acid	0.9500	0.048	0.001
L-Methionine	0.9592	0.050	0.004
Heptadecane	0.9000	0.086	0.004
2-Ketoisocaproic acid	0.9111	0.065	0.003
D-Glucuronic acid	0.9750	0.032	0.001
Propionic acid	0.8778	0.081	0.006
Anthracene	0.8000	0.113	0.040
Acetic Acid	0.9625	0.043	0.001
Estrone	0.9683	0.040	0.002
Glutamine	0.8857	0.081	0.008
Pyruvic acid	0.9889	0.018	0.000
D-Ribose	0.8778	0.088	0.006
Iron	0.9000	0.074	0.003
Sulfurous acid	0.9571	0.045	0.002
Caprylic acid	0.9571	0.045	0.002
Naphthalene	0.9286	0.077	0.010
Butyric acid	0.9667	0.051	0.011
Glutaconic acid	0.8929	0.086	0.011
Benzoic acid	0.9111	0.065	0.003
1,2-Benzenedicarboxylic acid	0.9250	0.081	0.013
L-Ascorbic acid	0.8056	0.153	0.090
cis-9-Hexadecenoic acid	0.9500	0.048	0.001
Hexadecanoic acid	0.8222	0.097	0.018
Inositol	0.9136	0.068	0.003
Octadecanoic acid	0.9222	0.077	0.002
Eicosapentaenoic acid	0.9889	0.018	0.000
Petroselinic Acid	0.9667	0.035	0.001
Myristic acid	0.8556	0.088	0.009
Cholesterol	0.9778	0.028	0.000
Adipic acid	0.8667	0.082	0.007

Table S2. Pathway analysis results of differential metabolites with MetaboAnalyst 5.0.

	Total	Expected	Hits	Raw *p*	-log(*p*)	Holm adjust	FDR	Impact
Aminoacyl-tRNA biosynthesis	48	0.80516	6	9.24E-05	4.0344	0.0077602	0.0055173	0.16667
Glyoxylate and dicarboxylate metabolism	32	0.53677	5	0.0001314	3.8815	0.010903	0.0055173	0.14815
Alanine, aspartate and glutamate metabolism	28	0.46968	4	0.0009706	3.013	0.078617	0.020382	0.16426
Glycine, serine and threonine metabolism	33	0.55355	4	0.0018317	2.7371	0.14471	0.025644	0.46284
Citrate cycle (TCA cycle)	20	0.33548	3	0.003956	2.4027	0.30461	0.041538	0.13471
Pyruvate metabolism	22	0.36903	3	0.0052261	2.2818	0.39718	0.048777	0.26749
Ascorbate and aldarate metabolism	8	0.13419	2	0.0071231	2.1473	0.53424	0.059834	0.5
Cysteine and methionine metabolism	33	0.55355	3	0.01638	1.7857	1	0.12509	0.1263
Fatty acid biosynthesis	47	0.78839	3	0.04168	1.3801	1	0.25008	0.01473
Glycolysis / Gluconeogenesis	26	0.43613	2	0.068738	1.1628	1	0.38494	0.1295
Inositol phosphate metabolism	30	0.50323	2	0.088342	1.0538	1	0.43651	0.12939
Sulfur metabolism	8	0.13419	1	0.12684	0.89673	1	0.56078	0.31915
Valine, leucine and isoleucine degradation	40	0.67097	2	0.14321	0.84401	1	0.6015	0.01084
Tyrosine metabolism	42	0.70452	2	0.15495	0.80981	1	0.6198	0.02463
Primary bile acid biosynthesis	46	0.77161	2	0.17897	0.74722	1	0.68334	0.05823
Pentose and glucuronate interconversions	18	0.30194	1	0.26376	0.5788	1	0.96328	0.125
Phosphatidylinositol signaling system	28	0.46968	1	0.3799	0.42033	1	1	0.03736
Glutathione metabolism	28	0.46968	1	0.3799	0.42033	1	1	0.08873
Steroid hormone biosynthesis	85	1.4258	2	0.42225	0.37443	1	1	0.04955
Steroid biosynthesis	42	0.70452	1	0.51332	0.28961	1	1	0.0282
Metabolism of xenobiotics by cytochrome P450	68	1.1406	1	0.69154	0.16018	1	1	0.0102

Table S3. The list of metabolic pathways.

Metabolic pathway ID	Metabolism	Metabolism ID	Metabolic pathway
A	Carbohydrate metabolism	1	Glycolysis / Gluconeogenesis
A	Carbohydrate metabolism	2	Citrate cycle (TCA cycle)
A	Carbohydrate metabolism	3	Pentose phosphate pathway
A	Carbohydrate metabolism	4	Pentose and glucuronate interconversions
A	Carbohydrate metabolism	5	Galactose metabolism
A	Carbohydrate metabolism	6	Ascorbate and aldarate metabolism
A	Carbohydrate metabolism	7	Amino sugar and nucleotide sugar metabolism
A	Carbohydrate metabolism	8	Pyruvate metabolism
A	Carbohydrate metabolism	9	Glyoxylate and dicarboxylate metabolism
A	Carbohydrate metabolism	10	Propanoate metabolism
A	Carbohydrate metabolism	11	Butanoate metabolism
A	Carbohydrate metabolism	12	C5-Branched dibasic acid metabolism
A	Carbohydrate metabolism	13	Inositol phosphate metabolism
B	Energy metabolism	14	Oxidative phosphorylation
B	Energy metabolism	15	Methane metabolism
B	Energy metabolism	16	Nitrogen metabolism
B	Energy metabolism	17	Sulfur metabolism
C	Lipid metabolism	18	Fatty acid biosynthesis
C	Lipid metabolism	19	Fatty acid elongation
C	Lipid metabolism	20	Fatty acid degradation
C	Lipid metabolism	21	Cutin, suberine and wax biosynthesis
C	Lipid metabolism	22	Steroid biosynthesis
C	Lipid metabolism	23	Primary bile acid biosynthesis
C	Lipid metabolism	24	Steroid hormone biosynthesis
C	Lipid metabolism	25	Glycerophospholipid metabolism
C	Lipid metabolism	26	Sphingolipid metabolism
C	Lipid metabolism	27	Biosynthesis of unsaturated fatty acids
D	Nucleotide metabolism	28	Purine metabolism
D	Nucleotide metabolism	29	Pyrimidine metabolism
E	Amino acid metabolism	30	Alanine, aspartate and glutamate metabolism
E	Amino acid metabolism	31	Glycine, serine and threonine metabolism
E	Amino acid metabolism	32	Cysteine and methionine metabolism
E	Amino acid metabolism	33	Valine, leucine and isoleucine degradation
E	Amino acid metabolism	34	Valine, leucine and isoleucine biosynthesis
E	Amino acid metabolism	35	Lysine biosynthesis
E	Amino acid metabolism	36	Arginine biosynthesis
E	Amino acid metabolism	37	Arginine and proline metabolism
E	Amino acid metabolism	38	Tyrosine metabolism
E	Amino acid metabolism	39	Phenylalanine metabolism
F	Metabolism of other amino acids	40	beta-Alanine metabolism
F	Metabolism of other amino acids	41	Taurine and hypotaurine metabolism
F	Metabolism of other amino acids	42	Phosphonate and phosphinate metabolism
F	Metabolism of other amino acids	43	Cyanoamino acid metabolism

Table S3. *Cont.*

Metabolic pathway ID	Metabolism	Metabolism ID	Metabolic pathway
F	Metabolism of other amino acids	44	D-Amino acid metabolism
F	Metabolism of other amino acids	45	Glutathione metabolism
G	Metabolism of cofactors and vitamins	46	Thiamine metabolism
G	Metabolism of cofactors and vitamins	47	Riboflavin metabolism
G	Metabolism of cofactors and vitamins	48	Vitamin B6 metabolism
G	Metabolism of cofactors and vitamins	49	Nicotinate and nicotinamide metabolism
G	Metabolism of cofactors and vitamins	50	Pantothenate and CoA biosynthesis
G	Metabolism of cofactors and vitamins	51	Lipoic acid metabolism
G	Metabolism of cofactors and vitamins	52	Porphyrin metabolism
H	Other metabolism	53	Biosynthesis of alkaloids derived from shikimate pathway
H	Other metabolism	54	Biosynthesis of alkaloids derived from ornithine, lysine and nicotinic acid
H	Other metabolism	55	Biosynthesis of alkaloids derived from histidine and purine
H	Other metabolism	56	Carbon metabolism
H	Other metabolism	57	2-Oxocarboxylic acid metabolism
H	Other metabolism	58	Biosynthesis of amino acids
H	Other metabolism	59	Biosynthesis of cofactors
H	Other metabolism	60	Glucosinolate biosynthesis
H	Other metabolism	61	Biosynthesis of various alkaloids
H	Other metabolism	62	Cholesterol metabolism
H	Other metabolism	63	Aminoacyl-tRNA biosynthesis
H	Other metabolism	64	Degradation of aromatic compounds
H	Other metabolism	65	Polycyclic aromatic hydrocarbon degradation
H	Other metabolism	66	Biosynthesis of nucleotide sugars
H	Other metabolism	67	Benzoate degradation
H	Other metabolism	68	Lipid metabolism
H	Other metabolism	69	Aminobenzoate degradation
H	Other metabolism	70	Fatty acid metabolism
H	Other metabolism	71	Monobactam biosynthesis
H	Other metabolism	72	Nucleotide metabolism

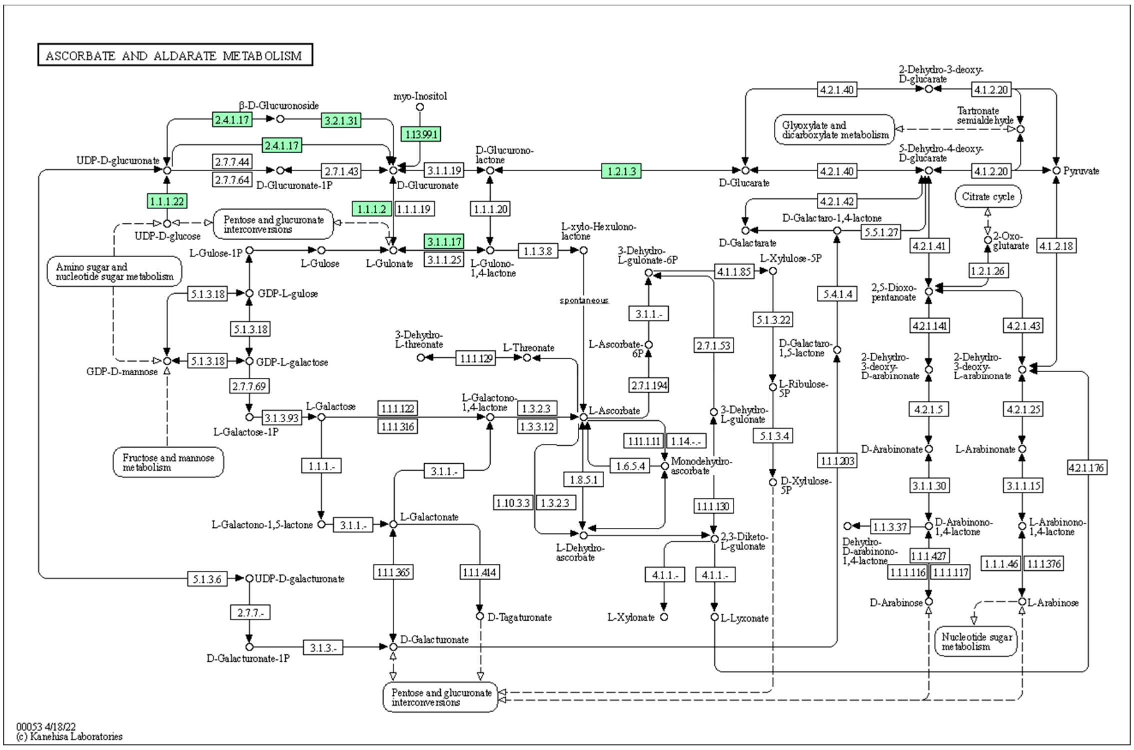

Figure S1. Ascorbate and aldarate metabolic pathway in adipose tissue of rats among different groups.
